# Depression and quality of life in schizophrenia-spectrum

**DOI:** 10.1192/j.eurpsy.2021.1373

**Published:** 2021-08-13

**Authors:** N. Smaoui, S. Elleuch, M. Maalej Bouali, S. Omri, R. Feki, N. Charfi, L. Zouari, J. Ben Thabet, M. Maalej

**Affiliations:** Psychiatry C Department, Hedi chaker University hospital, sfax, Tunisia

**Keywords:** schizophrénia, Depression, quality of life

## Abstract

**Introduction:**

The coming out of depressive disorders seems to be associated with severity of schizophrenia’s disease and with poor quality of life (QoL).

**Objectives:**

The aim of our study was to assess the relationship between depression and QoL in patients with schizophrenia.

**Methods:**

This is a cross-sectional and analytical study including stabilized patients with schizophrenia or schizoaffective disorder followed up in the outpatient psychiatry department at Hedi Chaker hospital university of Sfax (Tunisia), between August and October 2019. We used the Calgary Depression Scale (CDS) to evaluate depression and the Quality of Life Enjoyment and Satisfaction Questionnaire (Q-LES-Q-SF) to assess QoL.

**Results:**

We recruited 37 patients with a mean age of 49.14 years and a sex ratio of 4.66. Seventy-three (73%) of patients were followed for schizophrenia and 27% for schizoaffective disorder. They were married in 43.2% and 35.1% of patients had a regular work. According to CDS, 18.9% of patients had depression with a mean score of 2.27 (SD 2). QLESQSF mean score was 65.51. Depression was negatively correlated with Quality of Life Enjoyment and Satisfaction (r=-0.59, p<0.001). We did not find a significant difference in depression according to the socio-demographic characteristics of the respondents or the clinical features of the disease.

**Conclusions:**

It is clear that depression in patients with schizophrenia is associated with significant functional disability. Strategies to overcome the burden of depression may instil hope for functional recovery.

